# Exploring the predictive potential of programmed death ligand 1 expression in healthy organs and lymph nodes as measured by ^18^F-BMS-986192 PET: pooled analysis of data from four solid tumor types

**DOI:** 10.1136/jitc-2024-008899

**Published:** 2024-06-17

**Authors:** Iris H C Miedema, Johanna E. E. Pouw, Anne Kwakman, Gerben J C Zwezerijnen, Marc C Huisman, Florentine E F Timmer, Rieneke van de Ven, Tanja D de Gruijl, Geke A P Hospers, Adrianus J de Langen, C Willemien Menke-van der Houven van Oordt

**Affiliations:** 1 Department of Medical Oncology, Amsterdam UMC Locatie VUmc, Amsterdam, The Netherlands; 2 Imaging and Biomarkers, Cancer Centre Amsterdam, Amsterdam, The Netherlands; 3 Department of Radiology and Nuclear Medicine, Amsterdam UMC Location VUmc, Amsterdam, The Netherlands; 4 Otolaryngology / Head and Neck Surgery, Amsterdam UMC - Locatie VUMC, Amsterdam, The Netherlands; 5 Cancer Biology and Immunology, Cancer Centre Amsterdam, Amsterdam, The Netherlands; 6 Medical Oncology, University Medical Centre Groningen, Groningen, The Netherlands; 7 NKI, Amsterdam, The Netherlands

**Keywords:** Immune Checkpoint Inhibitor, fMRI / PET, Immune related adverse event - irAE, Biomarker

## Abstract

**Introduction:**

Immune checkpoint inhibitors (ICIs) can elicit anticancer immune responses, but predictive biomarkers are needed. We measured programmed death ligand 1 (PD-L1) expression in organs and lymph nodes using ^18^F-BMS-986192 positron emission tomography (PET)-imaging and looked for correlations with response and immune-related adverse events.

**Methods:**

Four ^18^F-BMS-986192 PET studies in patients with melanoma, lung, pancreatic and oral cancer, receiving ICI treatment, were combined. Imaging data (organ standardized uptake value (SUV)_mean_, lymph node SUV_max_) and clinical data (response to treatment and incidence of immune-related adverse events) were extracted.

**Results:**

Baseline PD-L1 uptake in the spleen was on average higher in non-responding patients than in responders (spleen SUV_mean_ 16.1±4.4 vs 12.5±3.4, p=0.02). This effect was strongest in lung cancer, and not observed in oral cancer. In the oral cancer cohort, benign tumor-draining lymph nodes (TDLNs) had higher PD-L1 uptake (SUV_max_ 3.3 IQR 2.5-3.9) compared with non-TDLNs (SUV_max_ 1.8, IQR 1.4-2.8 p=0.04). Furthermore, in the same cohort non-responders showed an increase in PD-L1 uptake in benign TDLNs on-treatment with ICIs (+15%), while for responders the PD-L1 uptake decreased (−11%). PD-L1 uptake did not predict immune-related adverse events, though elevated thyroid uptake on-treatment correlated with pre-existing thyroid disease or toxicity.

**Conclusion:**

PD-L1 PET uptake in the spleen is a potential negative predictor of response to ICIs. On-treatment with ICIs, PD-L1 uptake in benign TDLNs increases in non-responders, while it decreases in responders, potentially indicating a mechanism for resistance to ICIs in patients with oral cancer.

**WHAT IS ALREADY KNOWN ON THIS TOPIC**

- Whole-body programmed death ligand 1 (PD-L1) positron emission tomography (PET) imaging is an increasingly popular alternative for measuring PD-L1 expression in tumors. For treatment with immune checkpoint inhibitors (ICIs), tumor uptake of the anti-PD-L1 tracer ^18^F-BMS-986192 potentially has predictive properties in lung cancer (Niemeijer *et al*; Nat Commun., 2018) and melanoma patients (Nienhuis *et al*; JNM, 2022). A larger study (N=80, lung cancer) is currently recruiting in order to further evaluate these findings (NCT03564197).
- This field has been primarily focused on tumor uptake, while lymphoid organs are pivotal for the initiation of the tumor-immune response, and immune-related adverse events in healthy tissues often occur on treatment. Since standard procedures for PET scans always include whole body (head to mid-thigh) images, PD-L1 PET scans could reveal an important source of information that has so far been unexplored.

**WHAT THIS STUDY ADDS**

- To the best of our knowledge, this is the first study investigating the uptake of ^18^F-BMS-986192 in healthy organs and lymph nodes, and its correlation to response and immune-related adverse events. In our patient cohort of 47 patients across four solid tumor types, PD-L1 tracer uptake in the spleen was higher in non-responding patients than in responders, providing a potential negative predictor for response to ICIs. Interestingly, this effect was strongest in the lung cancer cohort, and not observed in the oral cancer cohort. Furthermore, in the oral cancer cohort benign tumor-draining lymph nodes in non-responding patients showed a significant increase in PD-L1 tracer uptake on treatment with ICI, while responding patients showed a decrease. Immune-related adverse events could not be predicted by PD-L1 PET uptake, but an increase in thyroid uptake on-treatment matched with either thyroid toxicity or a history of thyroid disease.

**HOW THIS STUDY MIGHT AFFECT RESEARCH, PRACTICE OR POLICY**

- PD-L1 PET uptake in the spleen is a potential negative indicator for response to immune checkpoint inhibition. This finding might be related to a state of systemic immunosuppression pretreatment driven by PD-L1 expression on splenic immune cells such as myeloid regulatory cells, providing an intriguing hypothesis for further research into the underlying mechanism. Similarly, the increased PD-L1 PET uptake in tumor-draining lymph nodes of patients with non-responding oral cancer on-treatment is most likely originating from antigen-presenting myeloid regulatory cells in the paracortex and might indicate a mechanism of resistance to ICIs. Larger trials (such as the aforementioned lung cancer study), could prove relevant for understanding these findings in relation to resistance to ICI and aiding treatment decisions.

## Introduction

Immune checkpoint inhibitors (ICIs) can reinvigorate anticancer immunity and have been shown to improve overall survival in various tumor types.^1–7^ Despite these favorable results, not all patients benefit and debilitating immune-related adverse effects (irAEs) may occur. Selecting those patients that will benefit from ICI treatment the most could improve the quality of care as well as suppress costs.

Currently, the most widely applied predictive biomarker for ICIs is immunohistochemical (IHC) analysis of programmed death ligand 1 (PD-L1), the ligand of programmed death 1 (PD-1), on tumor biopsies.^8^ Although this biomarker is used in clinical practice on a daily basis, it has multiple disadvantages such as the need for an invasive biopsy and the inability to assess interlesion or intralesion heterogeneity.^9^ As an attractive alternative, whole-body PD-L1 expression can be non-invasively assessed by virtue of positron emission tomography (PET) imaging, using the adnectin-based human anti-PD-L1 tracer ^18^F-BMS-986192.^10^ Previous studies in patients with non-small cell lung cancer (NSCLC) and metastatic melanoma have demonstrated the potential of ^18^F-BMS-986192 PET imaging as a predictive biomarker for treatment with ICIs.^11 12^ These studies have focused on PD-L1 tracer uptake in tumor tissues; however, these PET images also harbor information on the uptake in lymph nodes and lymphoid organs, which play a vital role in the initiation of a tumor-immune response and can reflect tumor-induced systemic effects.^3 13^ Moreover, PD-L1 tracer uptake in organs that are frequently affected by irAEs, such as the thyroid, liver or the pituitary gland, might reveal important insights into the development of immunotherapy-induced toxicities.^14^

In this study, the PD-L1 expression in healthy organs and lymph nodes was assessed by using available data from four different ^18^F-BMS-986192 PET imaging studies, totaling 75 scans across four solid tumor types: NSCLC, melanoma, pancreatic ductal adenocarcinoma (PDAC), and oral cavity squamous cell carcinoma (OCSCC). The association of organ and lymph node PD-L1 PET uptake with treatment response was explored, as well as potential correlations to the development of irAEs.

## Methods

### Data collection

**Figure 1:**
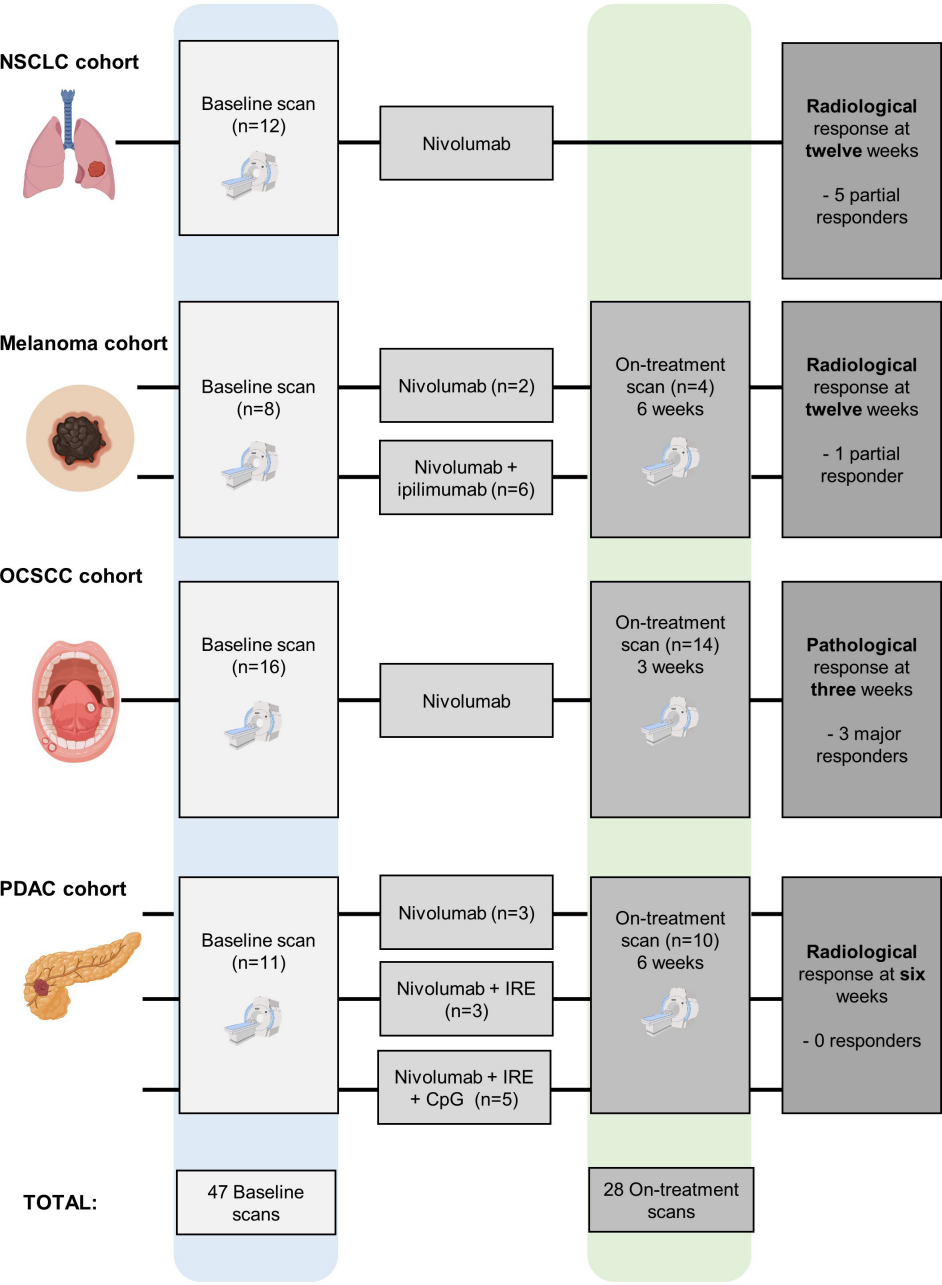
Flow diagram summarizing the data included in this manuscript. In total 75 scans from four tumor types were included. All patients received therapy with nivolumab. Some patients received additional treatment (ipilimumab (n=6), IRE (n=3), IRE+injection with CpG (n=5)). Figure is created with BioRender.com. CpG, cytosine-phosphate-guanine motifs, a toll-like receptor ligand, IRE, irreversible electroporation; NSCLC, non-small cell lung cancer; OCSCC, oral cavity squamous cell carcinoma; PDAC, pancreatic ductal adenocarcinoma.

Data from four PET-imaging studies using the anti-PD-L1 tracer ^18^F-BMS-986192 were combined: a study in patients with NSCLC by Niemeijer *et al*,^11^ a study in patients with melanoma by Nienhuis *et al*,^12^ the NeoNivo trial in patients with OCSCC^15^ and the PANFIRE III trial in patients with PDAC^16^. Details can be found in table 1 and figure 1.

**Table 1:** Overview of included ^18^F-BMS-986192 imaging studies

Malignancy	ClinicalTrials.gov ID	Characteristics	Key inclusion and exclusion criteria	Relevant study procedures	Ref.
NSCLC	NCT03520634	N=13 7 male Median age 63 (58–69)	Inclusion: advanced NSCLC, EGFR wild type and EML4-ALK fusion-negative, ECOG 0–1. Exclusion: symptomatic central nervous system metastases, use of corticosteroids,* interstitial lung disease or active infection.	Baseline: ^18^F-BMS-986192 PET/CT scans (N=13). On-treatment: not performed. Injected activity: 1.5–3.0 MBq/kg. Treatment: nivolumab 3 mg/kg, Q2W. Response evaluation: RECIST V.1.1 at 12 weeks.	11
Melanoma	NCT03520634	N=8 5 male Median age 62	Inclusion: stage IV metastatic melanoma, ECOG 0–1. Exclusion: autoimmune disease, treatment with immunosuppressive medication.	Baseline: ^18^F-BMS-986192 PET/CT scans (N=8). On-treatment: ^18^F-BMS-986192 PET/CT scans at 6 weeks (N=4). Injected activity: 185 MBq. Treatment: nivolumab (3 mg/kg) with (N=5) or without (N=3) ipilimumab (1 mg/kg), Q3W. Response evaluation: RECIST V.1.1 at 12 weeks.	12
OCSCC	NCT03843515	N=16 11 male Median age 72 (49–86)	Inclusion: stage III/IV resectable OCSCC, ECOG 0–1. Exclusion: secondary malignancy, use of corticosteroids,* interstitial lung disease or active infection.	Baseline: ^18^F-BMS-986192 PET/CT scans (N=16). On-treatment: ^18^F-BMS-986192 PET/CT scans at 3 weeks (N=14). Injected activity: 216 MBq (range 167–246). Treatment: nivolumab (single flat dose 480 mg). Response evaluation: pathological response.	Preliminary data,^15^ manuscript in preparation
PDAC	NCT01939665	N=11 5 male Median age 63 (43–75)	Inclusion: stage IV metastatic PDAC, max. five metastases of ≥1 cm, stable disease after FOLFIRINOX treatment, WHO 0–2. Exclusion: brain metastases, active autoimmune disease requiring corticosteroids* active infections, prior immunotherapy, secondary malignancy.	Baseline: ^18^F-BMS-986192 PET/CT scans (N=11). On-treatment: ^18^F-BMS-986192 PET/CT scans at 6± weeks (N=5). Injected activity: 3.0 MBq/kg (range 2.3–3.6). Treatment: nivolumab (cycle 1 and 2,240 mg, from cycle 2 and onwards 480 mg Q4W). Induction therapy with either (nivolumab 240 mg (N=3), IRE (N=3), or IRE and intratumoral CpG injection (N=3). Response evaluation: RECIST V.1.1 at 6 weeks, every 3 months.	Protocol,^16^ manuscript in preparation

*10 mg prednisone equivalent or more.

CpG, cytosine-phosphate-guanine motifs, a toll-like receptor ligand; CTLA-4, cytotoxic T-lymphocyte associated protein 4; ECOG, Eastern Cooperative Oncology Group performance status; EGFR, epidermal growth factor receptor; EML4-ALK, echinoderm microtubule-associated protein-like 4-anaplastic lymphoma kinase; IRE, irreversible electroporation; irPC, immune-related pathological response criteria; NSCLC, non-small cell lung cancer; OCSCC, oral cavity squamous cell carcinoma; PD-1, programmed death 1; PDAC, pancreatic adenocarcinoma; PD-L1, programmed death ligand 1; PET, positron emission tomography; Q2W, 2 weekly dosing; Q3W, 3 weekly dosing; RECIST, response evaluation criteria in solid tumors; WHO, WHO performance status.

### ^18^F-BMS-986192 biodistribution analysis

^18^F-BMS-986192 PET scans were acquired and reconstructed according to the EARL1 criteria (without point-spread function modeling),^17^ and analyzed using the in-house developed BIODISTRIBUTION tool (developed in IDL Virtual Machine Application V.8.4). For the OCSCC, melanoma and PDAC cohort, ^18^F-BMS-986192 tracer uptake in healthy tissues (brain, lungs, kidneys, liver, gall bladder, spleen) was acquired based on automatic pre-segmentations by an in-house developed artificial intelligence tool (courtesy of Professor Dr Boellaard), with manual corrections.^18^ In case of misalignment between ^18^F-BMS-986192 PET and CT scan, the volume of interest (VOI) only included the region that was overlapping between both scans. The thyroid was manually delineated with a VOI on each side of the trachea on three adjacent 4 mm CT slices axially oriented, with as cranial boundary the thyroid cartilage and as caudal boundary the apex of the lungs. Tonsils were delineated on three adjacent 4 mm CT slices at the back of the tongue. To determine the uptake in the bone marrow, fixed size VOIs were placed centrally in three adjacent lumbar vertebrae. The pituitary gland was localized on the CT scan at the base of the sella turcica and a VOI was manually drawn and if needed adjusted based on the PET uptake. Tracer uptake in the blood pool was measured using fixed size VOIs centrally placed in the aortic arch on five adjacent 4 mm axial CT slices. Assuming homogenous tracer uptake in the organs, tracer uptake in the organs is expressed as mean standardized uptake value (SUV_mean_): mean activity concentration normalized for body weight and injected dose. For the melanoma cohort, data was supplied from the University Medical Center Groningen and added to the database for analysis, methods for delineation are detailed in the respective publication.^12^

### ^18^F-BMS-986192 lymph node analysis and histology

Visual assessment of lymph nodes on PET/CT scan was performed by an experienced nuclear medicine physician (GJCZ). Cervical, axillary and inguinal lymph nodes with a short-axis diameter of at least 5 mm were identified using the Philips IntelliSpace Portal V.5 and SUV_max_ values were extracted. The SUV_max_ is used since it is less affected by the partial volume effect, that underestimates uptake in small regions, than the SUV_mean_ or SUV_peak_. Tumor-draining lymph nodes (TDLNs) were identified for the OCSCC cohort as the lymph nodes on the ipsilateral side of the tumor within the corresponding cervical lymph node level. TDLNs in the NSCLC and melanoma cohorts were not identifiable since no information on primary tumor lesion was available, and since the PDAC cohort TDLNs in de abdominal region could not be reliably delineated.

If possible, lymph nodes were matched to the pathology report based on their size and location. Histology was mostly available for the patients with OCSCC who underwent neck dissection, and one axillary lymph node from the NSCLC cohort. Lymph nodes that could not be matched to the pathology report, or for which no histology was available, were indicated as “not evaluated”.

### Response and adverse events evaluation

For the patients with OCSCC, response to treatment was measured as a pathological response score on post-treatment tumor resection material and scored by an experienced pathologist as % residual viable tumor (RVT).^19^ Response to treatment for the patients with melanoma, NSCLC and PDAC was defined according to the response evaluation criteria for solid tumors (RECIST) V.1.1 and determined by an experienced radiologist.^20^ In this study, the indication “response” was used to describe either a response according to RECIST V.1.1 (either partial or complete) or a major pathological response (≤10% RVT).^19 21^ In the respective studies, adverse events were monitored according to the National Cancer Institute Common Terminology for Adverse Events. For this study, we focused on organ-specific irAEs: thyroiditis, hypophysitis, nephritis, and hepatitis. In addition, the medical history of potential autoimmune thyroid disorders pretreatment was added.

### PD-L1 immunohistochemistry

PD-L1 IHC was performed on six lymph nodes from the OCSCC cohort. These cases were selected on the basis that, using the pathology report and PET imaging, they could be identified as the same lymph node. Staining was performed using a 22C3 clone on the DAKO Autolitic stainer, which was validated against the PharmDx kit.

### Statistical analyses

Statistical analyses were performed using R Statistical Software (V.4.2.1; R Foundation for Statistical Computing, Vienna, Austria). Tracer uptake in healthy organs was considered as normally distributed data. Differences in tracer uptake in healthy organs between baseline and follow-up PET scans were tested using a paired t-test. For normally distributed unpaired data, one-way analysis of variance or two-sided unpaired t-tests were performed. Tracer uptake in lymph nodes did not follow a normal distribution and was tested using the Kruskal-Wallis test for comparison between multiple groups, or with the Mann-Whitney U test. Normally distributed data (healthy organ uptake) was presented as mean with SD; not normally distributed data (lymph node uptake) was presented as median with first (Q_1_) and third quartile (Q_3_). A p value below 0.05 was considered statistically significant.

## Results

### Patient characteristics

75 ^18^F-BMS-986192 PET scans were reviewed from four solid tumor types: NSCLC (n=12),^11^ melanoma (n=12),^12^ OCSCC (n=30)^15^ (manuscript in preparation) and PDAC (n=21)^16^ (manuscript in preparation). For melanoma, OCSCC and PDAC, scans were available from baseline and on-treatment time points. For NSCLC, only baseline scans were available. Relevant information on patient characteristics and study procedures pertaining to the ^18^F-BMS-986192 PET scans can be found in table 1 and figure 1. For more detailed study procedures we refer to the individual publications.

### Baseline ^18^F-BMS-986192 uptake in healthy organs

**Figure 2:**
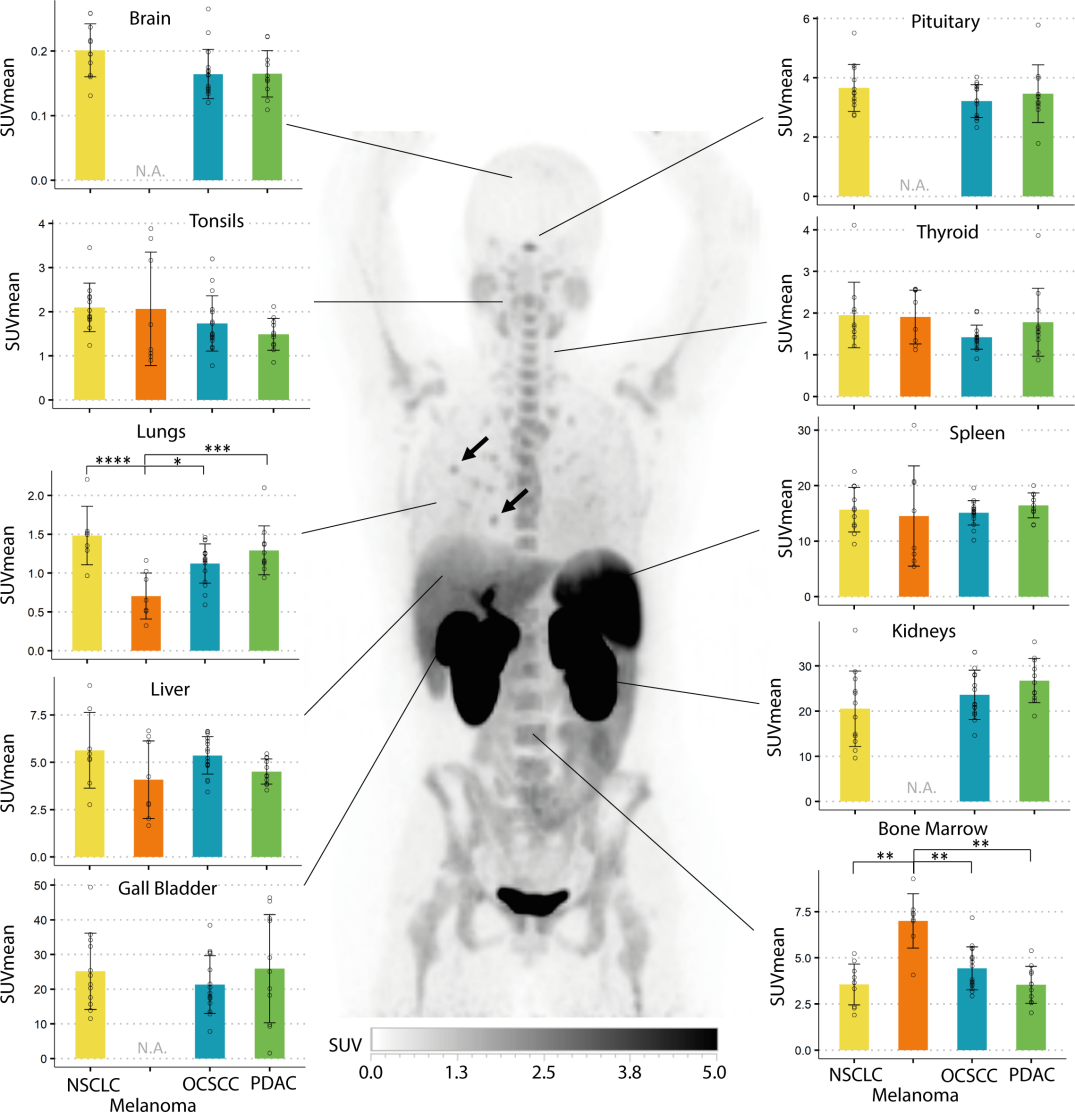
^18^F-BMS-986192 organ uptake at baseline. Representative ^18^F-BMS-986192 PET image of a patient with PDAC and quantification per organ at baseline (pretreatment). Mean, SD and individual data points are shown. Black arrows indicate examples of a high focal uptake observed in the lung. One-way analysis of variance results are shown if statistically significant (*p<0.05, **p<0.01, ***p<0.001, ****p<0.0001). NSCLC, non-small cell lung cancer; OCSCC, oral cavity squamous cell carcinoma; PDAC, pancreatic ductal adenocarcinoma; SUV, standardized uptake value.

Substantial uptake of ^18^F-BMS-986192 was noted in lymphoid organs, namely the spleen, liver, tonsils and bone marrow across all tumor types (figure 2). Lymph nodes showed varying ^18^F-BMS-986192 uptake and will be discussed in detail below. Patients with melanoma had a higher mean bone marrow uptake (SUV_mean_: 6.8±1.5) as compared with other tumor types (OCSCC SUV_mean_: 4.4±1.2, PDAC SUV_mean_: 3.5±1.0, and NSCLC SUV_mean_: 3.2±1.0). In addition to the liver, the gallbladder and kidneys also showed high uptake on the ^18^F-BMS-986192 PET scans, consistent with hepatobiliary and renal excretion of the tracer. The large variability in gallbladder uptake can partly be attributed to differences in the timing of food consumption and the resulting bile secretion, since no instructions regarding food consumption were given. Mean uptake in thyroid (SUV_mean_ 1.7±0.7) and pituitary gland (SUV_mean_ 3.4±0.8) was present at baseline. Surprisingly, focal areas with high tracer uptake were observed in the lungs of more than half of the reviewed ^18^F-BMS-986192 PET scans (black arrows in figure 2). No anatomical substrate underlying this focal PET uptake could be identified on the corresponding CT scans. Potentially, this local uptake can be due to tracer microemboli that have been described for ^18^F-FDG (online supplemental figure 1).^22 23^

10.1136/jitc-2024-008899.supp1 Supplementary data

**Figure 3:**
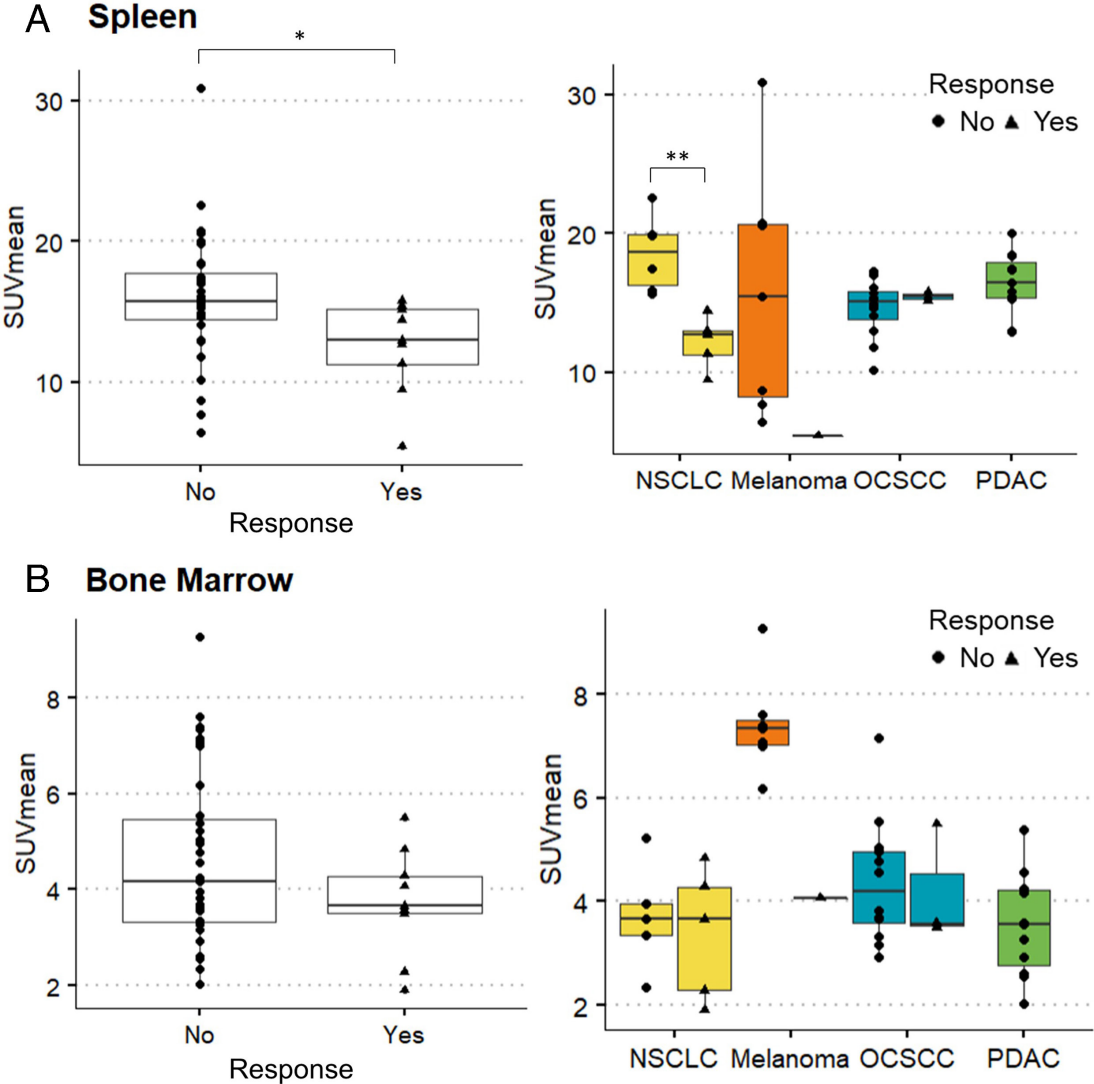
^18^F-BMS986192 spleen uptake at baseline is significantly lower in responding patients with NSCLC. (A) Spleen uptake of ^18^F-BMS986192 at baseline is higher in non-responding patients as compared with responding patients (SUV_mean_ 16.1±4.4 vs 12.5±3.4, p=0.02, two-sided t-test). Subanalysis shows this difference in NSCLC (SUV_mean_ 18.6±2.7 vs 12.2±1.9, p<0.01, two-sided t-test), and not in the OCSCC cohort. (B) For bone marrow uptake of ^18^F-BMS986192 a similar pattern was observed with higher uptake in non-responding patients as compared with responding patients, but this difference was not statistically significant (SUV_mean_ 4.6±1.8 vs 3.7±1.1, p=0.08, two-sided t-test). NSCLC, non-small cell lung cancer; OCSCC, oral cavity squamous cell carcinoma; PDAC, pancreatic ductal adenocarcinoma; SUV, standardized uptake value.

Spleen uptake of ^18^F-BMS986192 at baseline was on average higher in non-responding patients as compared with responding patients (SUV_mean_ 16.1±4.4 vs 12.5±3.4, p=0.02, two-sided t-test, figure 3A). Subanalysis showed this difference to be present in patients with NSCLC (SUV_mean_ 18.6±2.7 vs 12.2±1.9, p=0.001, two-sided t-test) and melanoma, but not in the OCSCC cohort. Bone marrow uptake of ^18^F-BMS-986192 followed a similar pattern with higher uptake in non-responding patients as compared with responding patients, but this difference was not statistically significant (bone marrow SUV_mean_ 4.6±1.8 vs 3.7±1.1, p=0.08, two-sided t-test, figure 3B). Uptake of ^18^F-BMS-986192 in liver and tonsils at baseline was not statistically different between responding and non-responding patients (online supplemental figure 2). Note that for the PDAC cohort no responses were observed.

10.1136/jitc-2024-008899.supp2 Supplementary data

### On-treatment ^18^F-BMS-986192 uptake in healthy organs

On-treatment data was available for patients with OCSCC, PDAC and melanoma. First, to enable comparison of tracer uptake between baseline and on-treatment, tracer clearance was compared between these time points by the SUV_mean_ in the aortic arch at the time of scanning. No statistically significant differences were found (online supplemental figure 3). Two patients of the PDAC cohort showed aberrantly increased tracer concentration in the aortic arch on-treatment compared with baseline, not related to patient characteristics or scanning parameters. Since uptake expressed in SUV is known to be heavily influenced by differences in plasma availability, these PET scans were excluded from the analysis. Second, we studied changes in uptake of ^18^F-BMS-986192 during ICI treatment, and we found that uptake was unchanged in most organs (online supplemental figure 4), with the exception of decreased tonsil uptake in patients with melanoma, and increased liver and bone marrow uptake on-treatment in patients with PDAC. Changes in ^18^F-BMS-986192 uptake between baseline and on-treatment scans did not correlate to response.

### Baseline ^18^F-BMS-986192 uptake in lymph nodes

A total of 201 lymph nodes were evaluated in patients with OCSCC, NSCLC and PDAC, from three regions: 56 cervical, 63 axillary and 82 inguinal. The pathological status (malignant/benign) was available for 42 lymph nodes (15 malignant/27 benign). 41 of them were from the patients with OCSCC, of whom most underwent surgical neck dissection. 1 LN with pathological status was retrived from a patient with NSCLC who underwent an axillary lymph node biopsy. The other 159 lymph nodes have not been biopsied (indicated as pathologically “not evaluated”); however, based on clinical staging and imaging these lymph nodes were not suspected to be malignant.

**Figure 4:**
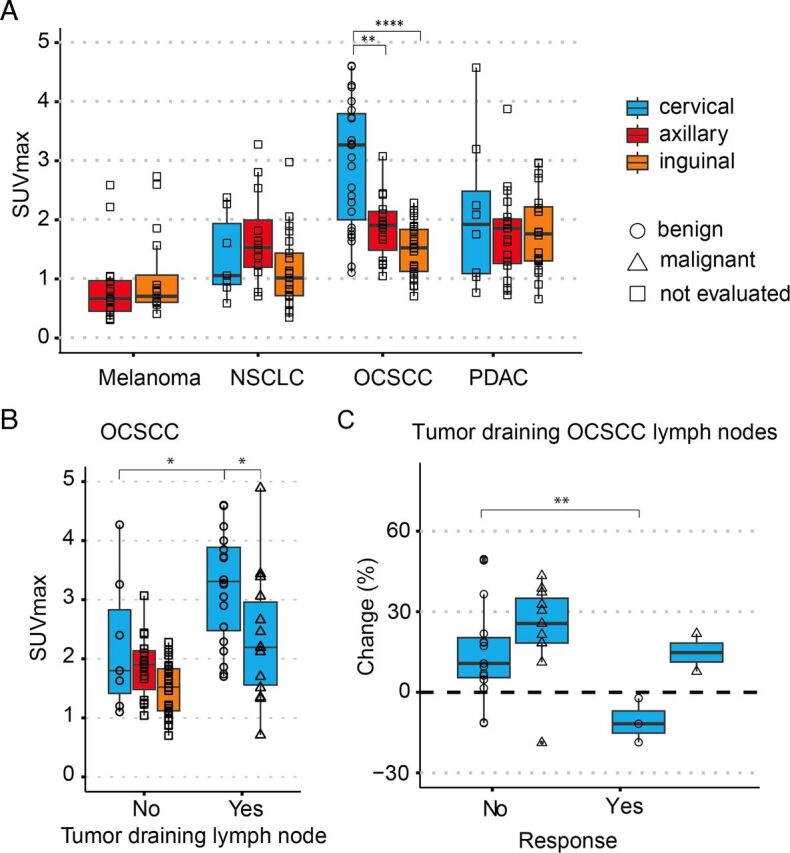
Baseline ^18^F-BMS-986192 uptake is increased in benign draining lymph nodes of patients with OCSCC. (A) SUV_max_ of non-malignant lymph nodes (benign+not evaluated) at baseline. A higher ^18^F-BMS-986192 uptake in the cervical lymph nodes of patients with OCSCC compared with their axillary and inguinal lymph nodes was seen (Kruskal-Wallis p=1.8e-06, Mann-Whitney U test with Bonferroni correction: cervical vs axillary, p<0.01, cervical vs inguinal p<0.0001). (B) In the OCSCC cohort benign TDLNs had significantly higher uptake than benign cervical lymph nodes that were not tumor draining (Mann-Whitney U test, p=0.04). In the tumor draining area, the uptake of benign lymph nodes was higher than of malignant lymph nodes (Mann-Whitney U test, p=0.02) in the same area. (C) Benign TDLNs in the OCSCC cohort showed an increase in ^18^F-BMS-986192 uptake (expressed as change (%)) from baseline to on-treatment in non-responders as compared with a decrease in ^18^F-BMS-986192 uptake in responders (mean change non-responders +15.2% ±18.4 vs mean change responders −10.8% ±8.3, p=0.009, Mann-Whitney U test). No difference was seen for non-TDLNs (online supplemental figure 7). NSCLC, non-small cell lung cancer; OCSCC, oral cavity squamous cell carcinoma; PDAC, pancreatic ductal adenocarcinoma; SUV, standardized uptake value; TDLN, tumor-draining lymph node.

Non-malignant lymph nodes are shown in figure 4A. Interestingly, ^18^F-BMS-986192 uptake in cervical lymph nodes of patients with OCSCC (SUV_max_ 3.26 IQR 2.00–3.79) was significantly higher than in axillary (SUV_max_ 1.90 IQR 1.48–2.14) or inguinal (SUV_max_ 1.52 IQR 1.12–1.83) lymph nodes. This effect was not apparent in patients with NSCLC or PDAC. Closer study of the OCSCC cohort, showed that this effect was mainly restricted to benign TDLNs, which had significantly higher ^18^F-BMS-986192 uptake (SUV_max_ 3.31 IQR 2.48–3.89) than benign non-TDLNs in the cervical region (SUV_max_ 1.80 IQR 1.42–2.83, p=0.04, Mann-Whitney U test) (figure 4B). Malignant TDLNs, had a significantly lower uptake of ^18^F-BMS-986192 (SUV_max_ 2.20 IQR 1.56–2.96) than the benign TDLNs (SUV_max_ 3.31 IQR 2.48–3.39, p=0.02, Mann-Whitney U test). Baseline ^18^F-BMS-986192 uptake in benign, malignant and non-evaluated lymph nodes was not related to response (online supplemental figure 5).

### On-treatment ^18^F-BMS-986192 uptake in lymph nodes

Across all cohorts, uptake of ^18^F-BMS-986192 in lymph nodes was increased on-treatment as compared with baseline, by an average of 21% (from median SUV_max_ 1.63–1.98, Wilcoxon signed-rank test, p=0.01, online supplemental figure 6). Interestingly, in benign TDLNs in the OCSCC cohort non-responders showed a significantly higher change (an increase of 15.2%) in ^18^F-BMS-986192 uptake as compared with responders (decrease of 10.8%) (figure 4C). In the contralateral non-TDLNs this effect was not seen (online supplemental figure 7), nor in the axillary or inguinal lymph nodes. Additionally, the change in uptake for melanoma and PDAC is shown in online supplemental figure 8.

### PD-L1 immunohistochemistry on cervical OCSCC lymph nodes

**Figure 5:**
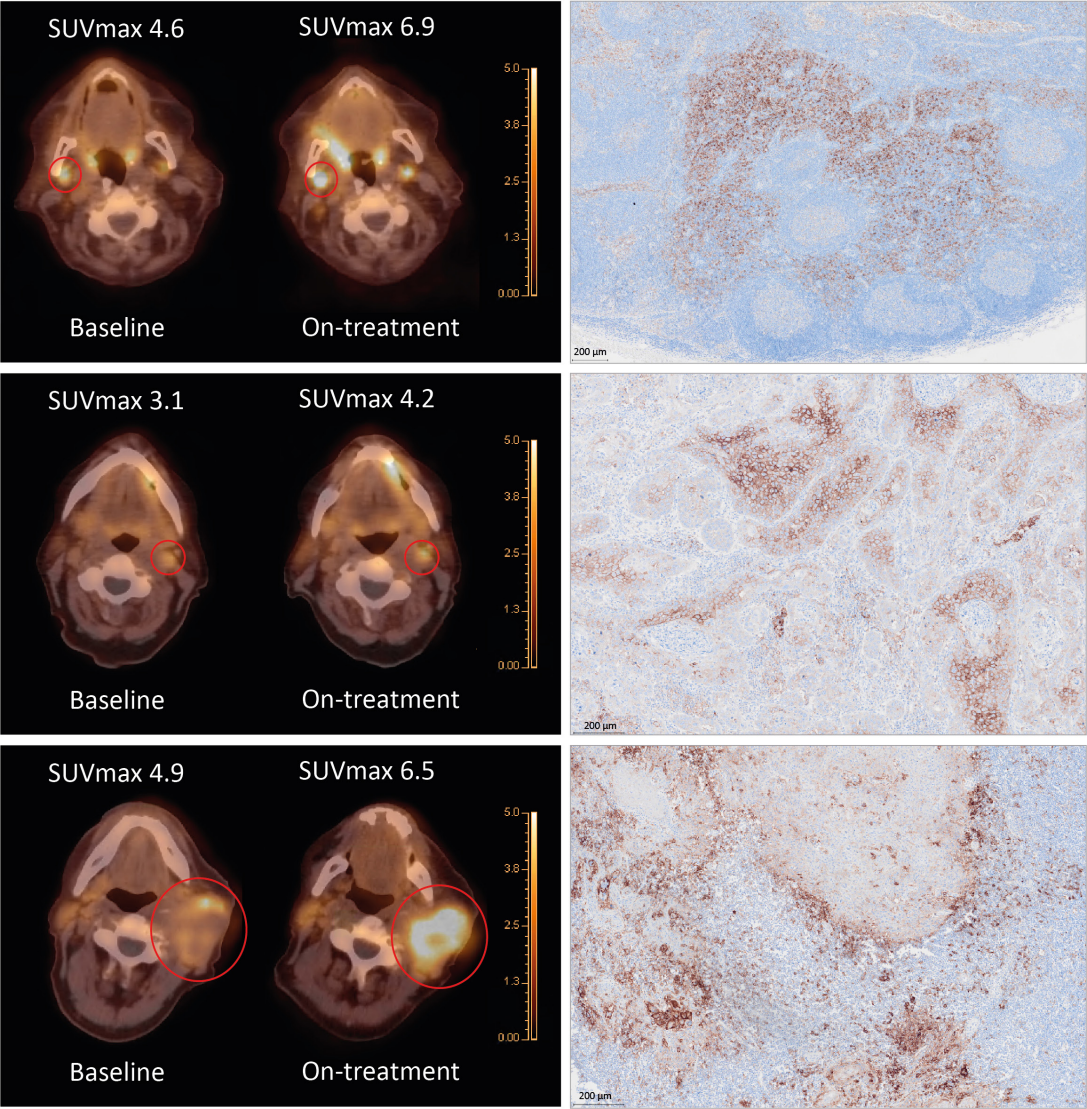
Examples of increases in ^18^F-BMS-986192 PET uptake in cervical lymph nodes of patients with OCSCC and matching PD-L1 immunohistochemistry staining. Upper panel: a benign tumor-draining lymph node with a ~50% increase in ^18^F-BMS-986192 uptake shows strong PD-L1 staining of macrophages in the paracortex covering ~30% of the total lymph node area. Middle panel: a malignant lymph node with a ~35% increase in ^18^F-BMS-986192 uptake with PD-L1 staining observed predominantly on tumor cells. Bottom panel: a large malignant lymph node with an increase in ^18^F-BMS-986192 uptake of ~33% shows PD-L1 staining is localized on macrophages, with tumor cells being completely PD-L1 negative. The PD-L1-positive macrophages seem to be concentrated around the tumor cell border. Note: fusion PET/CT axial images are shown, a red circle is placed around the indicated lymph node. FFPE tissues were used from the neck dissection performed after the on-treatment scan. These three patients were all non-responders. FFPE, formalin-fixed, paraffin-embedded PD-L1, programmed death ligand 1; PET, positron emission tomography.

PD-L1 IHC was performed on cervical lymph nodes from the OCSCC cohort that could be easily matched to the corresponding location on the PD-L1 PET scan. Three cases are highlighted in figure 5, demonstrating diverse patterns of PD-L1 staining. The first case shows a benign TDLN with a ~50% increase in ^18^F-BMS-986192 uptake following ICI treatment, with PD-L1 expression localizing predominantly to myeloid cells in the paracortex and covering ~30% of the total lymph node area. The middle panel is a malignant lymph node with a ~35% increase in ^18^F-BMS-986192 uptake following ICI treatment, with PD-L1 staining predominantly observed on tumor cells. The third demonstrates a large malignant lymph node with an increase in ^18^F-BMS-986192 uptake of ~33% following ICI treatment, with PD-L1 staining on cells with a myeloid phenotype, most likely macrophages. These PD-L1 positive macrophages concentrated around the tumor border, while the tumor cells were PD-L1 negative.

### ^18^F-BMS-986192 uptake in organs and correlation to irAEs

**Figure 6:**
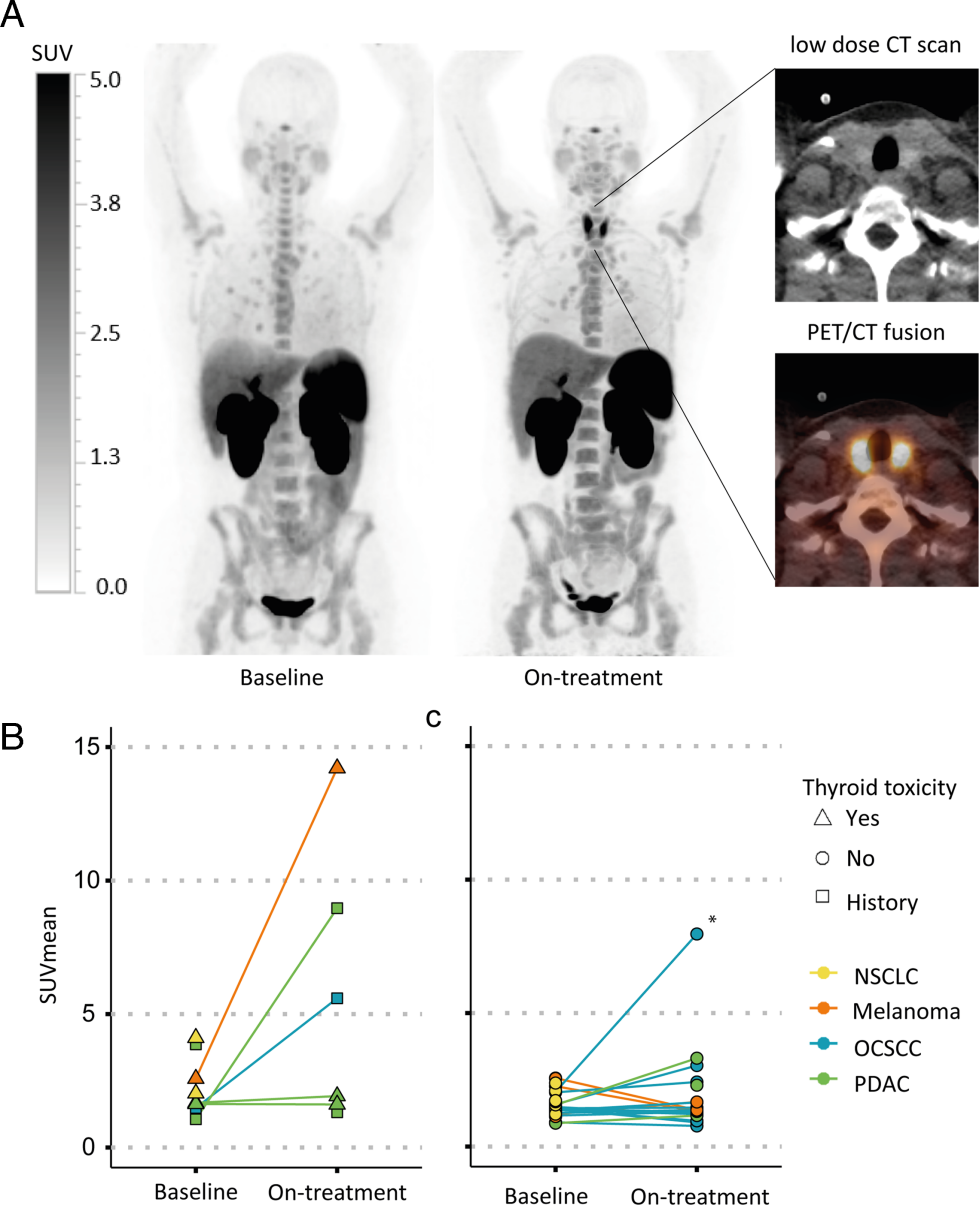
Increased thyroid uptake was observed in four patients on-treatment. (A) Maximum intensity projections (left) and axial CT and PET/CT fusion images (right) demonstrate an example of an increase in thyroid uptake on-treatment. SUV bar is shown. (B) Thyroid PD-L1 PET uptake in patients with either a history of thyroid disease or who experienced thyroid toxicity on-treatment. Three patients had a marked increase in thyroid PD-L1 uptake. (C) Thyroid PD-L1 PET uptake in patients without history of thyroid disease or thyroid toxicity on-treatment. Only one patient showed an increase in thyroid uptake on-treatment (indicated by the asterisk). Interestingly, this specific patient from the OCSCC cohort who had been treated with a single dose neoadjuvant nivolumab, developed hypothyroidism 3 months after concurrent chemoradiotherapy treatment. Baseline thyroid uptake could not predict the development of thyroid toxicity. NSCLC, non-small cell lung cancer; OCSCC, oral cavity squamous cell carcinoma; PDAC, pancreatic; PD-L1, programmed death ligand 1; PET, positron emission tomography; SUV, standardized uptake value.

Baseline ^18^F-BMS-986192 thyroid uptake was not predictive of the development of immune-related thyroid toxicity (figure 6). However, a remarkable increase in ^18^F-BMS-986192 thyroid uptake on-treatment as compared with baseline was seen in a total of four patients: two in the OCSCC, one in the melanoma and one in the PDAC cohort. Only one out of these four patients developed irAE thyroiditis. However, two patients had a history of either hyperthyroidism or hypothyroidism, and the third, who had been treated with a single dose of nivolumab according to study protocol, eventually developed hypothyroidism during concurrent chemoradiotherapy after 3 months. Conversely, three patients who developed immune-related thyroid toxicity on-treatment (two in the NSCLC cohort, one in the PDAC cohort) did not show higher uptake of ^18^F-BMS-986192 on either baseline or on-treatment PET scans.

Hepatitis was observed twice in the NSCLC cohort, once in the PDAC cohort and twice in the melanoma cohort. The average SUV_mean_ in the liver of these patients at baseline was not different from patients who did not develop hepatitis (SUV_mean_ 4.22±0.9 vs 5.06±1.5). Nephritis (grade 1) was observed in one patient in the PDAC cohort after two cycles of nivolumab, which was accompanied by a remarkably high kidney uptake at baseline (SUV_mean_ 35.4 vs overall average SUV_mean_ 23.6±6.7) and with a strong reduction on-treatment (SUV_mean_ 16.1 vs overall average SUV_mean_ 22.0±6.8). Central hypothyroidism was observed once in the melanoma cohort, but no delineation was performed on the pituitary gland. Other irAEs such as colitis, rash or myositis were not evaluated since these organs were not delineated in this study.

## Discussion

In this study, ^18^F-BMS-986192 PET biodistribution data of four tumor types (melanoma, NSCLC, OCSCC and PDAC) was studied in healthy organs and lymph nodes. High baseline PD-L1 PET uptake in the spleen is negatively associated with response to ICI treatment. Although further validation is needed, this signal seems especially strong for patients with metastatic NSCLC, and might ultimately serve as a negative predictive biomarker. The ongoing multicenter clinical trial using ^18^F-BMS-986192 PET imaging in 80 patients with NSCLC is perfectly suited for further evaluation of this signal (NCT03564197).

The relevance of PD-L1 PET uptake in the spleen within the context of ICI treatment, could be related to PD-L1 expression on myeloid regulatory cells (MRCs). By secreting soluble factors, cancer cells can condition myeloid cells at distant sites like the spleen and bone marrow to become MRCs, including M2-like macrophages and myeloid-derived suppressor cells (MDSCs).^24–26^ In addition, newly generated immature myeloid cells can be skewed to differentiate into an MDSC phenotype instead of a more favorable mature macrophage, dendritic cell or granulocyte phenotype.^27 28^ Since MRCs can express high levels of PD-L1,^29 30^ the PD-L1 PET uptake in the spleen could be a measure for the number of MRCs at the start of treatment, reflecting a state of systemic immune suppression and providing a negative predictor of outcome. Interestingly, previous studies using ^18^F-FDG PET describe a similar higher uptake of ^18^F-FDG in the spleen at baseline in patients with an unfavorable outcome to ICI treatment in metastatic melanoma.^31–33^ MRCs can also exhibit an altered glucose metabolism with a high rate of glycolysis, high glucose uptake, and consequently a high ^18^F-FDG uptake.^34^ Therefore, MRCs could be responsible for the increased ^18^F^−^FDG uptake in the spleen seen in these studies. It should be noted that high spleen uptake might be a pure prognostic biomarker and not specific for ICI treatment.

In future studies, the role of MRCs in lymphoid organs such as the spleen warrants further translational research. Specifically in the field of PET imaging, myeloid cell tracers like CD11b,^35^ CD163^36^ or the mannose receptor CD206^37^ could provide a more specific myeloid-derived signal. Alternatively, one study reported PD-L1 expression on CD8α+CD68+ endothelial cells, sharing phenotypic features with MRCs, lining the venous sinusoids of the spleen. How these cells causally relate to response to ICI treatment is currently unclear.^38^

In this study, we also quantified PD-L1 PET uptake in cervical, axillary and inguinal lymph nodes and found a significantly higher PD-L1 uptake in the cervical lymph nodes of the patients with OCSCC at baseline, highest in benign TDLNs. As a part of standard clinical staging, patients with OCSCC undergo fine needle aspiration cytology (FNAC) biopsies of neck lymph nodes. Whether the PD-L1 uptake in these benign TDLNs at baseline could have been elevated as a result of FNAC, or that the higher PD-L1 uptake is a response to soluble tumor factors, warrants further research. However, these benign TDLNs also behaved differently on-treatment with ICI, when no further biopsies were performed: non-responders had a significant increase of mean +15% in PD-L1 PET uptake on treatment with ICIs, whereas a decrease of mean −11% was seen in TDLNs of responding patients, potentially reflecting a local resistance mechanism. IHC staining showed PD-L1 expression in lymph nodes was either found on tumor cells, myeloid cells in the paracortex or tumor fields, or both. Therefore, in benign TDLNs, the high PD-L1 PET uptake is most likely linked to antigen-presenting cells such as macrophages or dendritic cells (DCs). On the one hand, PD-L1 expression on myeloid cells in the TDLN can be a sign of immune activation. While being an inhibitory ligand for PD-1, PD-L1 expression is induced on various myeloid cells as a result of activation and functions as a negative feedback mechanism to circumvent the overactivation of the adaptive immune response. For instance, DCs migrating to draining lymph nodes will often highly express PD-L1, as it has been linked with their migratory potential^39^ and also macrophages within lymph nodes express PD-L1 physiologically.^40^ On the other hand, PD-L1-expressing lymph node resident DCs have recently been shown to restrain antitumor T cells in the TDLNs^41^ and PD-L1 expression on antigen-presenting cells distant from any tumor metastases in TDLNs were shown to predict subsequent distant metastasis.^42^ These observations fit with our observation of higher PD-L1 PET uptake at baseline in TDLNs, and an on-treatment increase being related to ICI resistance. It will be important to study PD-L1 presence on myeloid cells together with markers of immune activation (CD80 in particular as it may neutralize PD-L1 by in-cis binding^43^) or immune suppression in order to further understand this signal. Interestingly, during treatment with ICIs, PD-L1 PET uptake increased by 21% across all tumor cohorts and lymph node locations and independent of response, indicating also a systemic reaction to the ICI.

In the four studied cohorts combined, only a limited number of irAEs occurred, hampering robust statistical evaluation. However, patients who showed an increase in thyroid PD-L1 PET uptake on-treatment could all be associated with either a history of thyroid disease or the development of thyroid toxicity on-treatment. On the contrary, not all patients who developed thyroid toxicity on-treatment had elevated PD-L1 PET uptake either at baseline or on-treatment scans. Nonetheless, an increase in PD-L1 expression might reflect a tendency for autoimmunity of the thyroid. Activation of the PD-1/PD-L1 pathway has been previously shown to play a role in autoimmune thyroid disease.^44^ For patients developing hepatitis, no differences in PD-L1 PET uptake in the liver were observed. Other irAEs (hypophysitis, nephritis) did not occur frequently enough to study correlation with PD-L1 PET uptake.

Typically, PET imaging studies include a limited number of subjects, as a result of factors like complexity of study procedures, scan duration, radiation burden, etc. In this study, by combining four imaging studies, we have tried to overcome this limitation. However, it is important to exercise caution when interpreting these combined results, for these trials have some inherent differences, for example, in the timing of on-treatment scan (3 weeks or 6 weeks), concomitant treatment and injected activity. Possibly, in the case of the baseline bone marrow and lung uptake (figure 2), differences in delineation techniques may have occurred between researchers (manual delineation vs pre-segmentation). Artificial intelligence tools that delineate PET scans can limit these types of differences as well as increase efficiency for researchers.^18^

Based on the full pharmacokinetic analysis of ^18^F-BMS-986192 performed in tumors of patient with NSCLC at baseline by Huisman *et al*,^10^ we used SUV normalized for body weight as our primary outcome measure. However, changes can occur on-treatment (eg, changes in metabolism) and the appropriate pharmacokinetic model and outcome measure may be affected. In our data set we did not find significant differences in the activity concentration in blood between baseline and on-treatment, and we expect SUV to be valid, but additional dynamic analysis on-treatment and in the organs are needed to confirm this.^10^

In conclusion, this study demonstrates that uptake of the anti-PD-L1 tracer ^18^F-BMS-986192 in spleen, especially in NSCLC, might be a potential predictive biomarker for response to ICI treatment, possibly related to the presence of MRCs. Furthermore, patients with OCSCC who did not respond to ICI treatment showed an increase in PD-L1 uptake in benign tumor-draining lymph nodes, whereas there was a decrease in responders. Finally, irAEs could not be predicted by baseline PD-L1 uptake; however, an increase in thyroid PD-L1 PET uptake on-treatment only occurred in patients with thyroid toxicity or a history of thyroid disease.

## Data Availability

Data are available upon reasonable request.
