# Supplementary material for: Exploring the predictive potential of programmed death ligand 1 expression in healthy organs and lymph nodes as measured by 18F-BMS-986192 PET: pooled analysis of data from four solid tumor types
Source: J Immunother Cancer. 2024 Jun 17;12(6):e008899. doi: 10.1136/jitc-2024-008899 (PMC11184194; doi:10.1136/jitc-2024-008899)
Supplement: Supplementary data [file jitc-2024-008899supp001.pdf]

BMJ Publishing Group Limited (BMJ) disclaims all liability and responsibility arising from any reliance placed on this supplemental material which has been supplied by the author(s)

Exploring the predictive potential of PD-L1 expression in healthy organs and lymph nodes as measured by ^{18}F-BMS986192 PET: pooled analysis of data from four solid tumor types

Supplemental figures

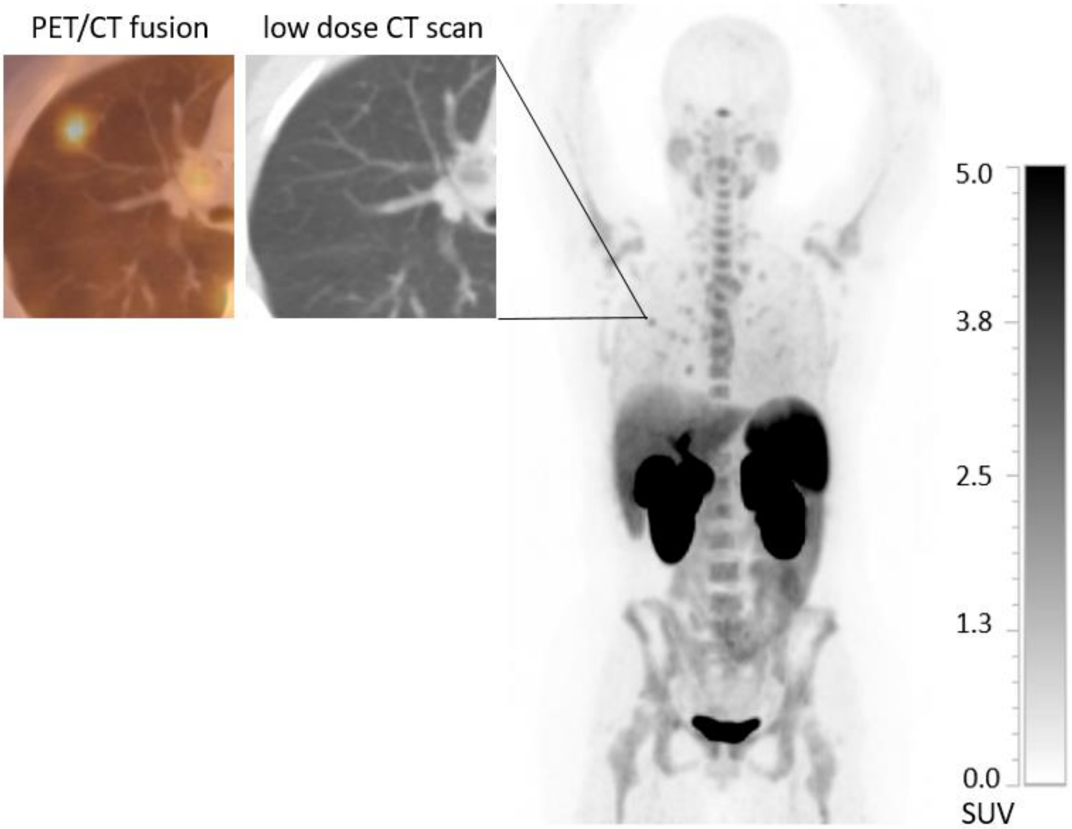

Supplemental Figure 1: ^{18}F-BMS-986192 PET images (maximum intensity projection) of a patient with PDAC at baseline. An example of an area with high focal tracer uptake in the right lung is highlighted (SUVmax 4.3). On the low dose CT scan no anatomical substrate was seen. Abbreviations: PDAC = pancreatic ductal adenocarcinoma, PET = positron emission tomography, CT = computed tomography).

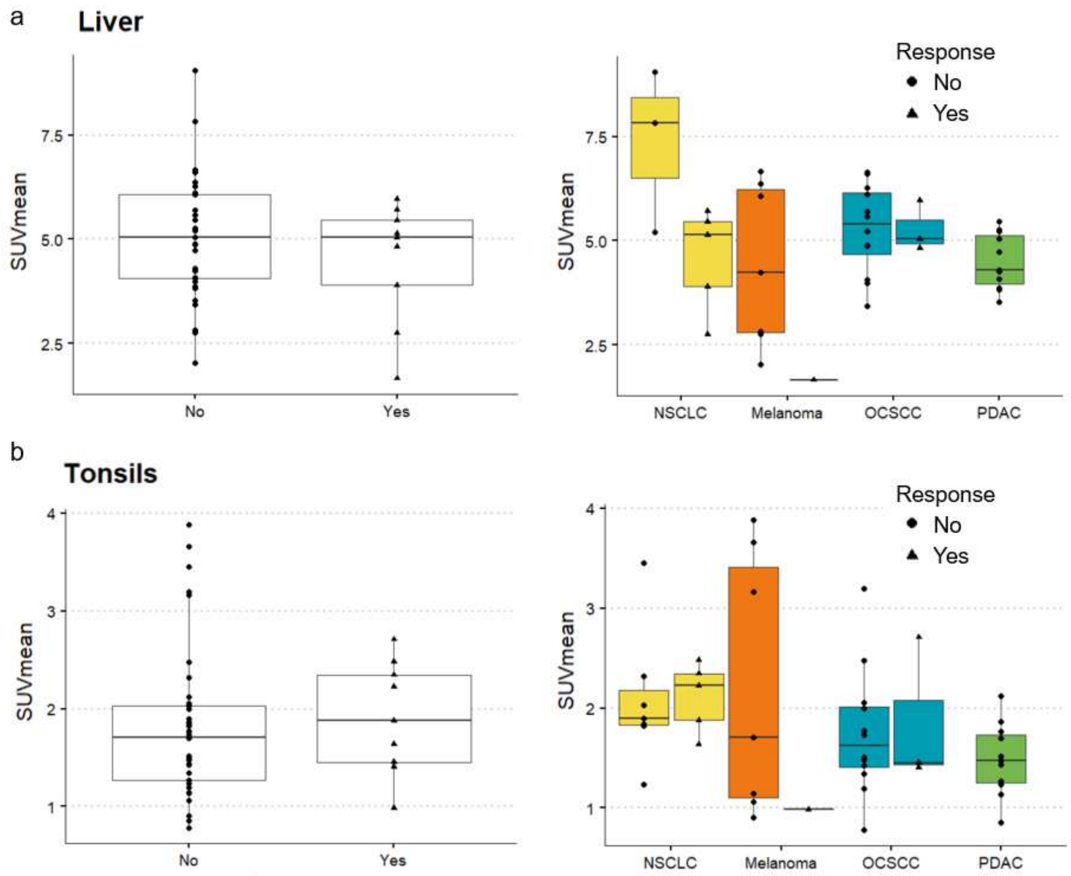

Supplemental Figure 2: liver and tonsil uptake in relation to response. ^{18}F-BMS-986192 uptake in liver and tonsils at baseline is not statistically different between non-responding and responding patients (Liver SUV_{mean} 5.0 ±1.5 vs. 4.5 ±1.4; p=0.3, Tonsils: SUV_{mean} 1.8 ±0.8 vs. 1.9 ±0.6; p=0.7, two-sided t-tests).

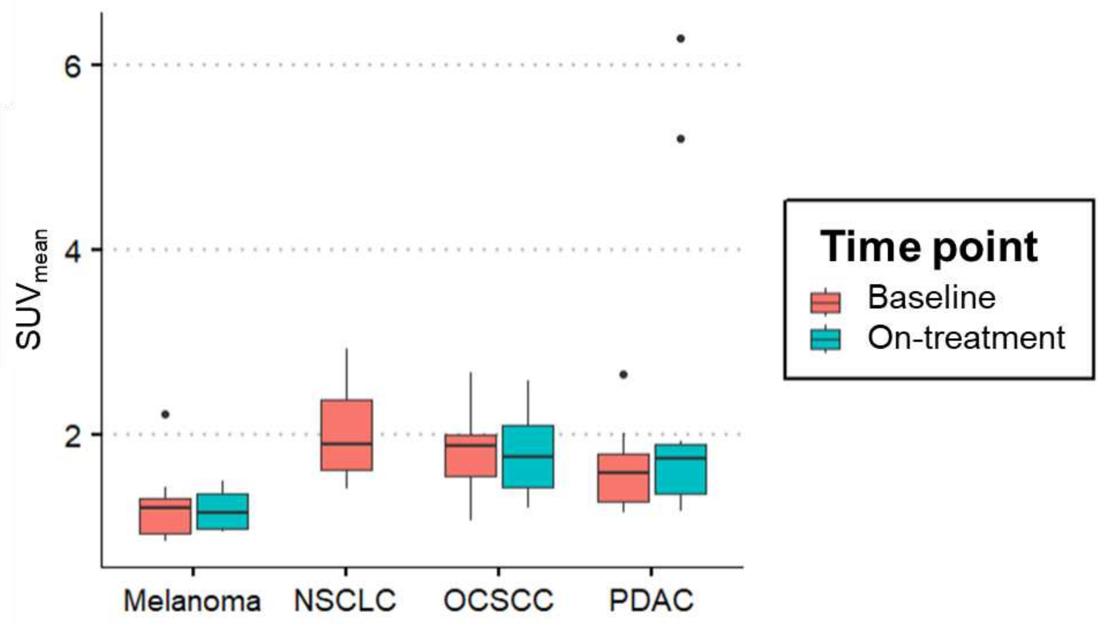

Supplemental Figure 3: Aortic arch tracer availability (SUV_{mean}) between baseline and on-treatment time points per tumor type. Note two major outliers in the PDAC on-treatment cohort, these two patients were excluded from further analysis. No statistically significant differences were observed between baseline and on-treatment uptake (Melanoma p=0.68, OCSCC p=0.89, PDAC p=0.48, Mann-Whitney U-tests).

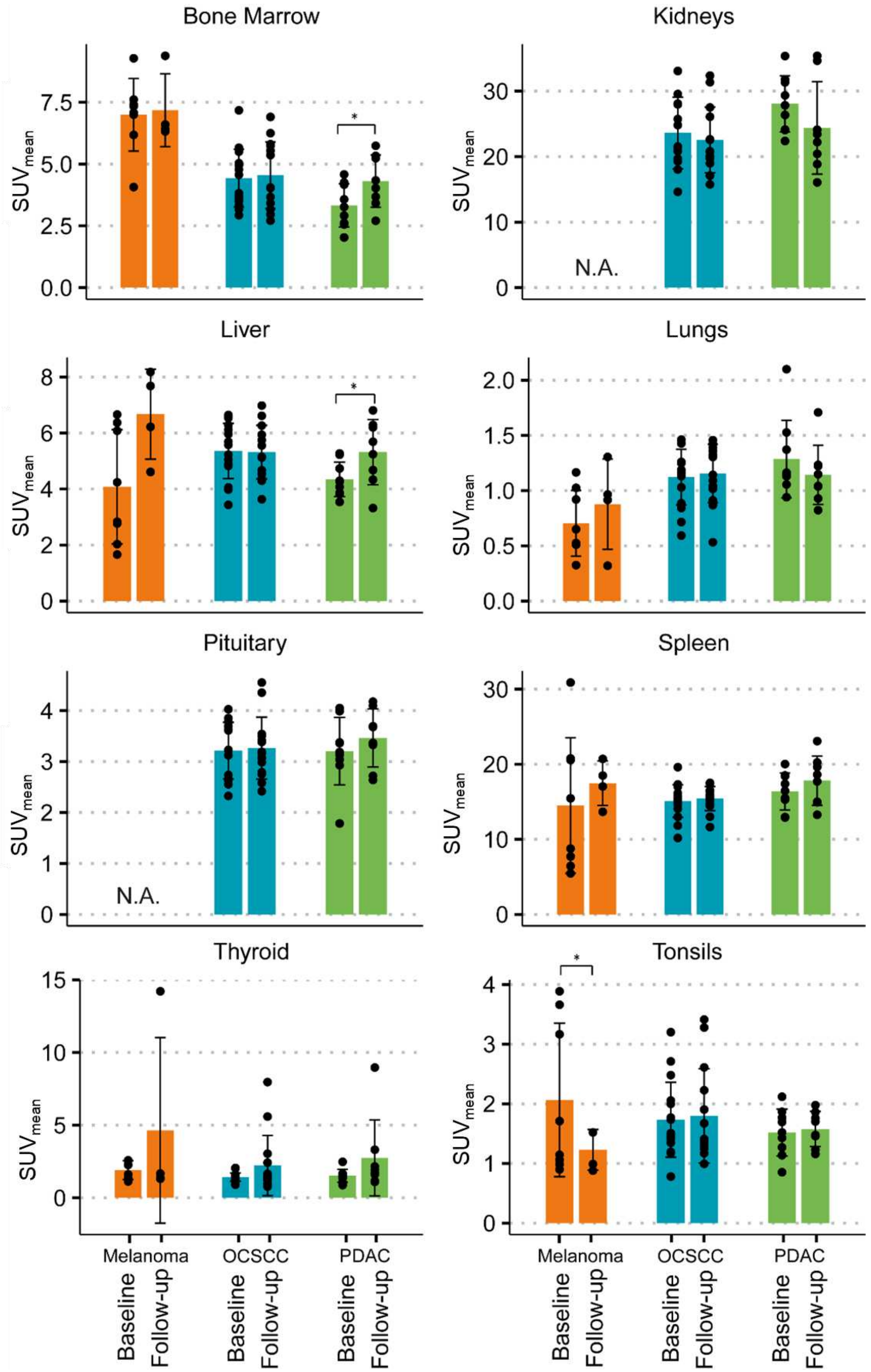
Bone Marrow
Kidneys
Liver
Lungs
Pituitary
Spleen
Thyroid
Tonsils
SUVmean
N.A.
Melanoma
OCSCC
PDAC
Baseline
Follow-up

30

Supplemental Figure 4: ^{18}F-BMS-986192 tracer uptake (SUV_{mean}) in healthy tissues at baseline and follow-up for patients with melanoma, OCSCC and PDAC. For patients with PDAC liver and bone marrow uptake were significantly higher on-treatment (PDAC liver: p=0.04, PDAC bone marrow: p=0.005, paired t-tests). Tonsil uptake has been reported as significantly higher at baseline than follow-up (p=0.002, [1]) for melanoma patients. In other organs no significant differences were observed. Mean and standard deviation are visualized per study and per scan time point. N.A. = not available.

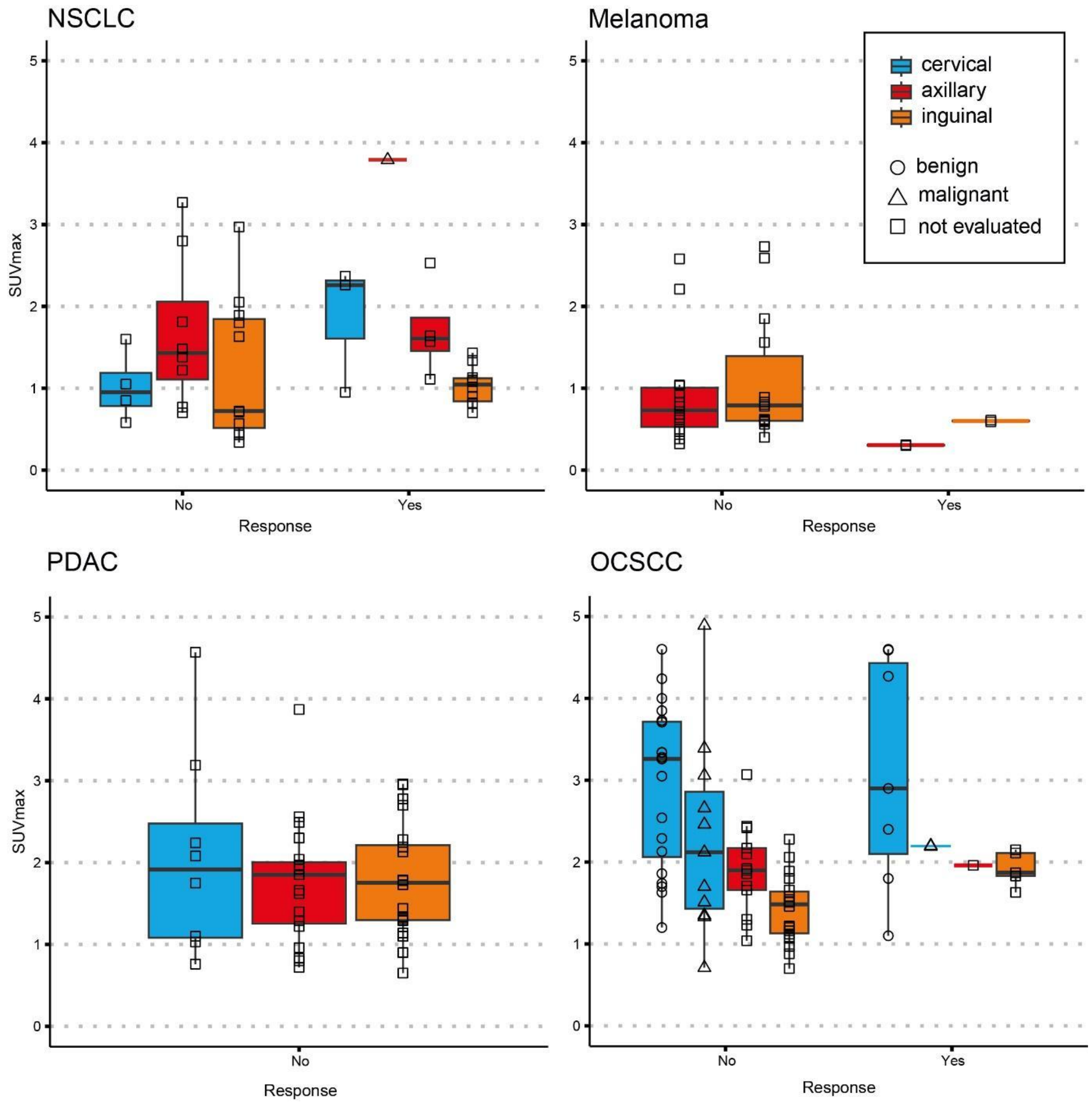

Supplemental Figure 5: baseline uptake of ^{18}F-BMS-986192 in lymph nodes as it relates to response to treatment. No correlation was found between SUV_{max} at baseline and response to treatment (Mann Whitney U-test).

BMJ Publishing Group Limited (BMJ) disclaims all liability and responsibility arising from any reliance placed on this supplemental material which has been supplied by the author(s)

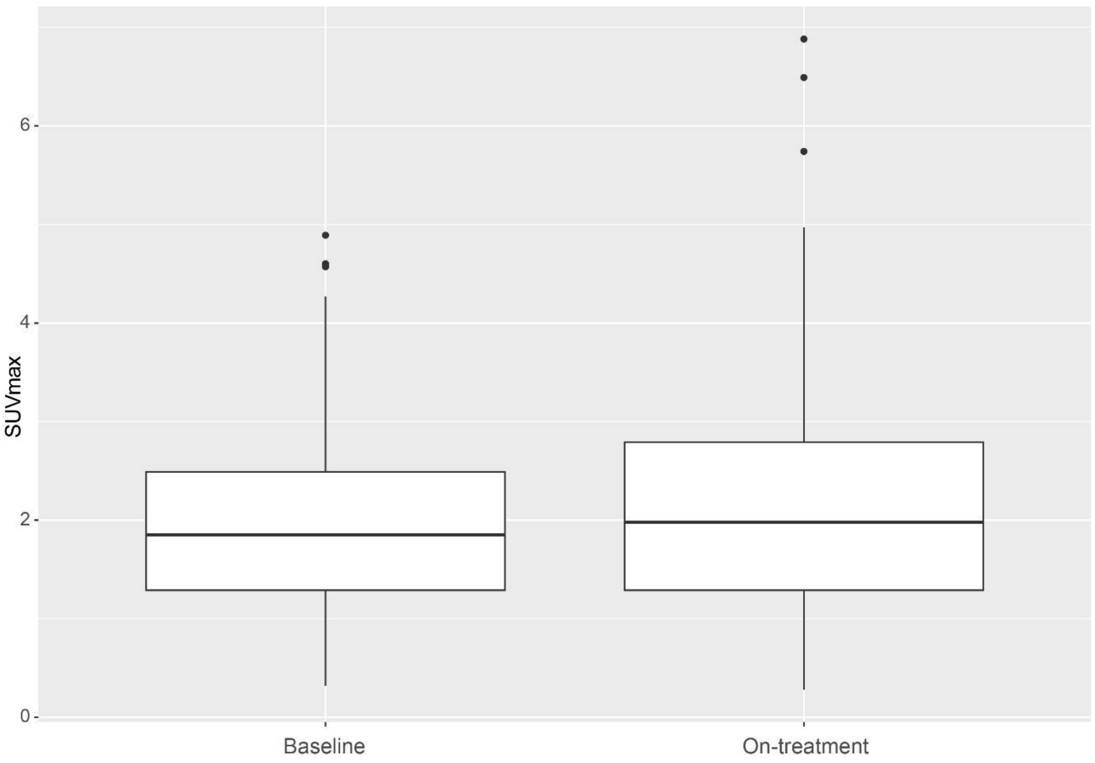

Supplemental Figure 6: overall uptake of ^{18}F-BMS-986192 in lymph nodes increases from baseline to on-treatment. Increase is shown from median SUV_{max} 1.63 to 1.98 (p=0.01, Wilcoxon signed rank test,).

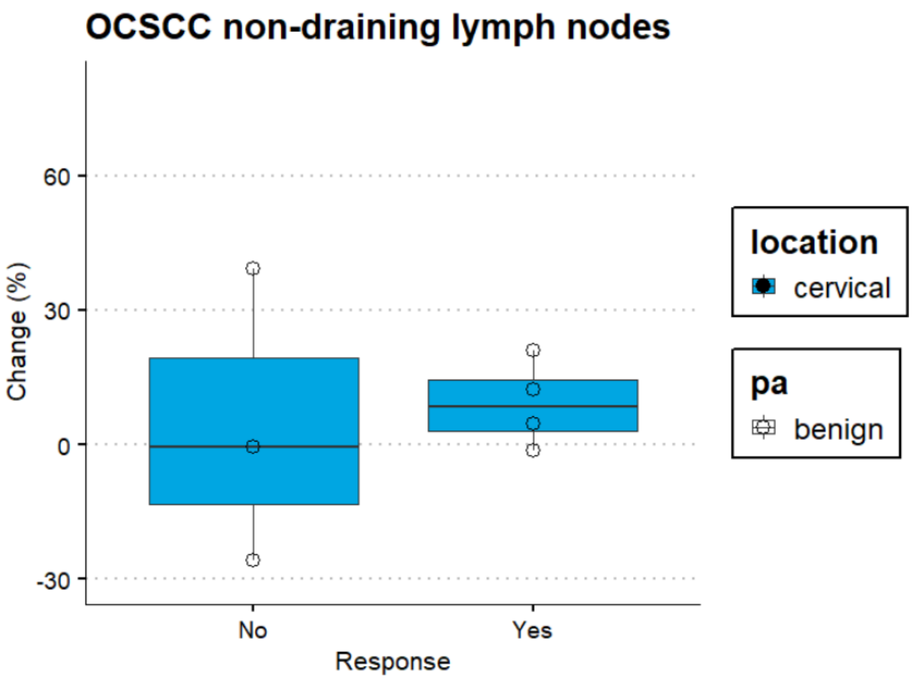

Supplemental Figure 7: change (%) in ^{18}F-BMS-986192 uptake from baseline to on-treatment. No statistically significant difference was seen between responders and non-responders in the non-draining cervical lymph nodes of OCSCC patients (Mann-Whitney U-test).

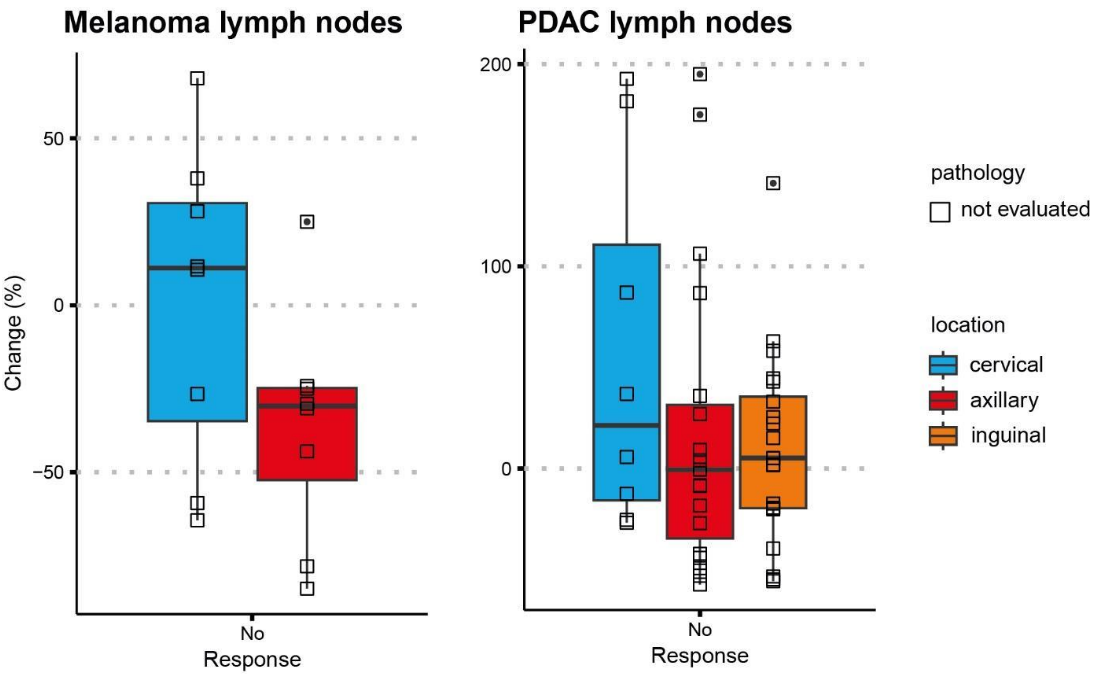

Supplemental Figure 8: change (%) in ^{18}F-BMS-986192 uptake from baseline to on-treatment in PDAC and melanoma cohort. Note that only non-responders were available for on-treatment assessment (there were no responders in the PDAC cohort, the responder in the melanoma cohort did not undergo follow-up scan).

References

1 Nienhuis, P. H. *et al.* (18)F-BMS986192 PET Imaging of PD-L1 in Metastatic Melanoma Patients with Brain Metastases Treated with Immune Checkpoint Inhibitors: A Pilot Study. *J Nucl Med* **63**, 899-905 (2022). https://doi.org:10.2967/jnumed.121.262368
