# Supplementary figures and images for: Exploring the predictive potential of programmed death ligand 1 expression in healthy organs and lymph nodes as measured by 18F-BMS-986192 PET: pooled analysis of data from four solid tumor types
Source: J Immunother Cancer. 2024 Jun 17;12(6):e008899. doi: 10.1136/jitc-2024-008899 (PMC11184194; doi:10.1136/jitc-2024-008899)

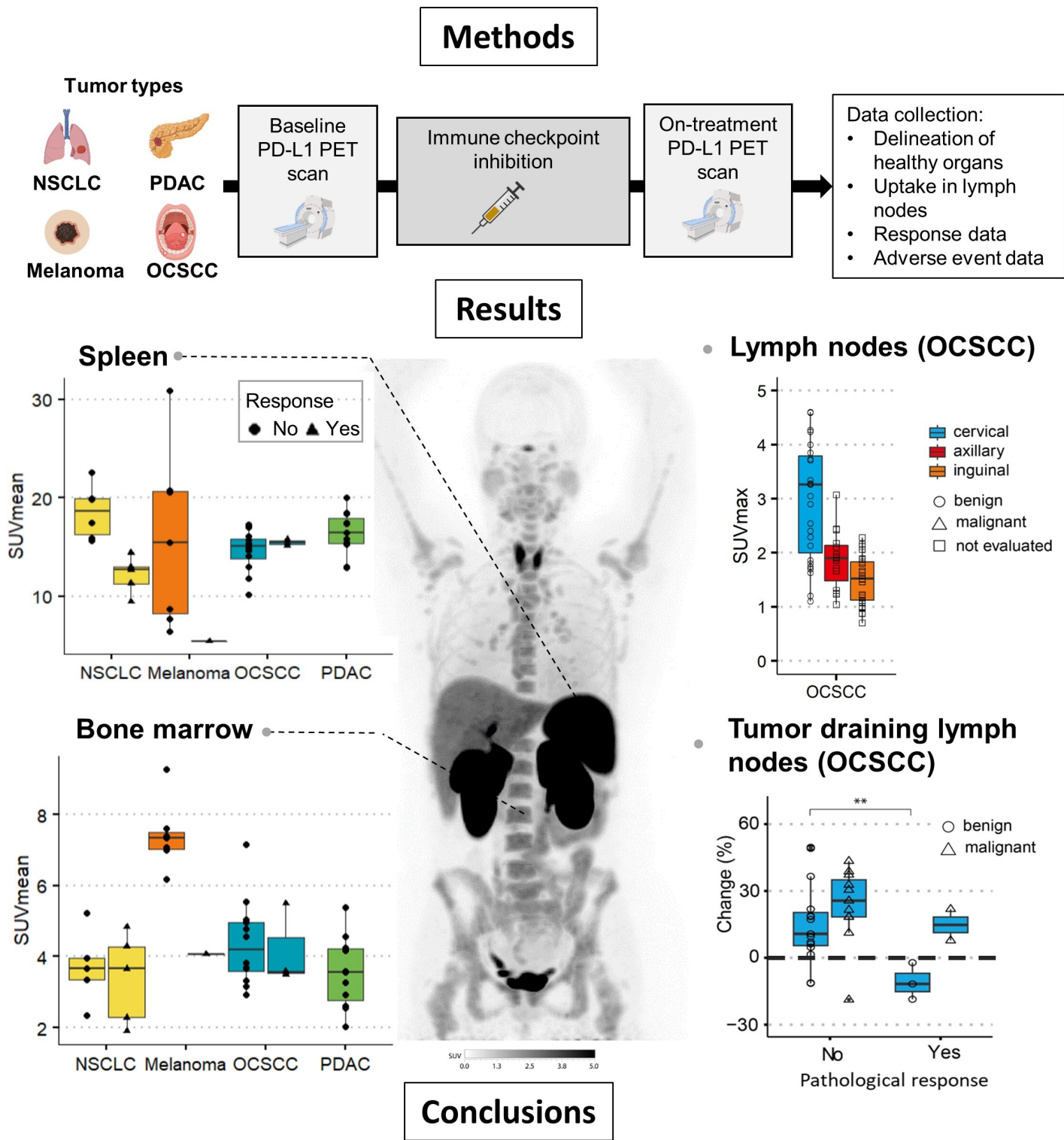

Supplement: Supplementary data [file jitc-2024-008899supp002.pdf]
